# Taking movement data to new depths: Inferring prey availability and patch profitability from seabird foraging behavior

**DOI:** 10.1002/ece3.3551

**Published:** 2017-10-25

**Authors:** Marianna Chimienti, Thomas Cornulier, Ellie Owen, Mark Bolton, Ian M. Davies, Justin M. J. Travis, Beth E. Scott

**Affiliations:** ^1^ School of Biological Sciences University of Aberdeen Aberdeen UK; ^2^ Marine Scotland Science Marine Laboratory Scottish Government Aberdeen UK; ^3^ RSPB Centre for Conservation Science North Scotland Office Inverness UK; ^4^ RSPB Centre for Conservation Science The Lodge Sandy Bedfordshire UK

**Keywords:** animal movements, foraging strategies, patch profitability, predators, prey availability

## Abstract

Detailed information acquired using tracking technology has the potential to provide accurate pictures of the types of movements and behaviors performed by animals. To date, such data have not been widely exploited to provide inferred information about the foraging habitat. We collected data using multiple sensors (GPS, time depth recorders, and accelerometers) from two species of diving seabirds, razorbills (*Alca torda*,* N *= 5, from Fair Isle, UK) and common guillemots (*Uria aalge*,* N *= 2 from Fair Isle and *N *= 2 from Colonsay, UK). We used a clustering algorithm to identify pursuit and catching events and the time spent pursuing and catching underwater, which we then used as indicators for inferring prey encounters throughout the water column and responses to changes in prey availability of the areas visited at two levels: individual dives and groups of dives. For each individual dive (*N *= 661 for guillemots, 6214 for razorbills), we modeled the number of pursuit and catching events, in relation to dive depth, duration, and type of dive performed (benthic vs. pelagic). For groups of dives (*N *= 58 for guillemots, 156 for razorbills), we modeled the total time spent pursuing and catching in relation to time spent underwater. Razorbills performed only pelagic dives, most likely exploiting prey available at shallow depths as indicated by the vertical distribution of pursuit and catching events. In contrast, guillemots were more flexible in their behavior, switching between benthic and pelagic dives. Capture attempt rates indicated that they were exploiting deep prey aggregations. The study highlights how novel analysis of movement data can give new insights into how animals exploit food patches, offering a unique opportunity to comprehend the behavioral ecology behind different movement patterns and understand how animals might respond to changes in prey distributions.

## 1 INTRODUCTION

Food resources are generally aggregated over a range of scales in hierarchical patch systems where high‐density patches at small scales are nested within low‐density patches at larger scales (Fauchald, Erikstad, & Skarsfjord, 2000; Regular, Hedd, & Montevecchi, 2013; Weimerskirch, Gault, & Cherel, 2005). Foragers should adjust their foraging patterns in such complex environments to maximize foraging efficiency. Optimal foraging theory (OFT) is widely used to explain the foraging behavior of animals. According to this theory, predators foraging in patchy environments make decisions to maximize the rate of energy intake while minimizing energy costs (Pyke, Pulliam, & Charnov, 1977; Stephens & Charnov, 1982). This relies on the assumption that predators adjust their time spent within a patch based on their expectation of locating a new and richer patch when they move on.

Technical advances over the last 50 years have enabled the collection of data which can capture the types of movement, feeding behavior, and physiological processes in environments where direct observations of behavior are difficult or impossible (Evans, Lea, & Patterson, 2013). In both marine and terrestrial environments, satellite transmitters, GPS, time–depth recorders (TDRs), and accelerometers have provided some of the most voluminous and informative data to date. The analysis and interpretation of these types of data involve the classification of different behavioral patterns, reconstructed time–depth profiles, and quantification of costs and benefits of different movement patterns (Brown, Kays, Wikelski, Wilson, & Klimley, 2013; Hays et al., 2016; Hussey et al., 2015; Kays, Crofoot, Jetz, & Wikelski, 2015).

A wide range of statistical methods facilitates behavioral classification of movement patterns (Bailey, Hammond, & Thompson, 2014; Bestley, Jonsen, Hindell, Guinet, & Charrassin, 2013; Bestley, Jonsen, Hindell, Harcourt, & Gales, 2015; Jonsen, Flemming, & Myers, 2005; Langrock et al., 2012; Morales, Haydon, Frair, Holsinger, & Fryxell, 2004; Pinto & Spezia, 2015). These methods show that foraging predators typically follow the hierarchical patchy distribution of resources varying their search tactics at several spatial and temporal scales. Predators may use hierarchical foraging tactics, using search patterns to maximize their chances of encountering prey aggregations (Fauchald et al., 2000; Fryxell et al., 2008; Pinaud, 2007). However, especially in the marine environment, questions such as how predators forage at fine scales, how they react to prey availability (e.g., densities and distributions), how they select and assess resources, and how they make decisions whether to stay or leave the foraging patch are poorly understood. Recently, studies have begun to associate movement data with the quantitative assessment of prey density and distribution (Boyd et al., 2015; Carroll et al., 2017; Goldbogen et al., 2015). Combining tracking data with prey survey data is crucial for investigating how predators relate to food resources and how they respond to changes in prey abundance and distribution. However, combining these data sources is costly and limited by the availability of spatially and temporally co‐occurring datasets.

For diving marine predators, especially marine mammals and seabirds, dive metrics such as dive duration, bottom duration, dive shape, descent, and ascent rate have been used to infer foraging activity and the quality of prey patches visited (Austin, Bowen, McMillan, & Iverson, 2006; Elliott et al., 2008; Machovsky Capuska, Vaughn, Würsig, Katzir, & Raubenheimer, 2011; Watanuki et al., 2006). Depending on species and search tactics, restricted search behaviors may be performed above or below water in association with persistent sea shelf oceanographic fronts and fine‐scale physical features such as horizontal and vertical currents which are known to create favorable foraging locations (Cox et al., 2016; Garthe, Montevecchi, Chapdelaine, Rail, & Hedd, 2007; Scales et al., 2014; Waggitt, Cavenave, Torres, Williamson, & Scott, 2016). In deep diving predators, such as marine mammals and penguins, it has been assumed that prey acquisition increases linearly with search time (Mori, 1998; Mori, Takahashi, Mehlum, & Watanuki, 2002; Thompson & Fedak, 2001). Based on this assumption, dive shapes have been classified and assigned to different behaviors: foraging, transiting, and resting (Thums, Bradshaw, & Hindell, 2008). The combination of multiple sensors recording different types of high‐frequency movements provides unique data which can be used to observe the behavior of tracked animals at different spatial and temporal scales. For example, a limited number of studies, where pursuit and catching events were recorded with cameras, have extracted more complex gain functions when comparing the number of prey caught with residence time in patches of prey species (e.g., Watanabe, Ito, & Takahashi, 2014). Moreover, recent analysis combining TDRs and accelerometers highlighted that the common interpretation that a longer bottom phase duration is an indication of higher foraging success may not always be accurate (Viviant, Jeanniard‐du‐Dot, Monestiez, Authier, & Guinet, 2016). It is important to use the correct metrics of foraging success to reflect true foraging success accurately (Aguilar Soto et al., 2008; Foo et al., 2016; Viviant, Trites, Rosen, Monestiez, & Guinet, 2010; Volpov, Hoskins, Battaile, & Viviant, 2015). The information acquired from combining data from multiple tracking devices not only has the potential to provide detailed pictures of the behaviors performed but can also be used as a tool to infer information indirectly about the environment that animals experience and how they might adjust foraging patterns in response to environmental variation (Guinet et al., 2014). Temporal variation in predator behavior is likely to provide insights into the spatial distribution of highly dynamic prey sources that may be difficult to track in other ways. With these new types of detailed information, there is a need for new methods to explore in more detail how to infer availability of food resources and the profitability of the habitat visited from a predator's perspective.

**Figure 1:**
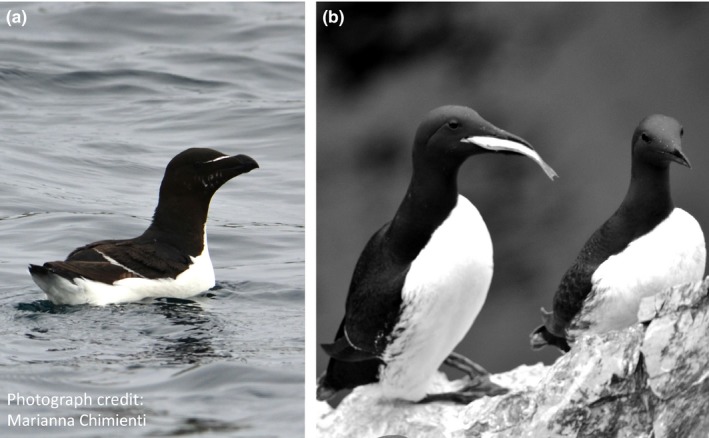
Photographs of (a) Razorbill (*Alca torda*) and (b) common guillemot (*Uria aalge*) taken by Marianna Chimienti

Diving seabird species, such as auks, use the water column for only a limited amount of time during foraging, making it more difficult to examine movements, search strategies, predator–prey interactions, and how foraging behavior relates to the surrounding habitat (Elliott et al., 2008; Doniol‐Valcroze et al., 2011). Common guillemots (*Uria aalge,* Figure 1) and razorbills (*Alca torda,* Figure 1) are wing‐propelled pursuit divers (Croll, Gaston, Burger, & Konnoff, 1992). Despite having the same diving method, these two species show differences in foraging behavior, diving to different depths and performing different activities underwater while chasing prey (Chimienti et al., 2016; Thaxter et al., 2010). When foraging, these two species typically fly between a series of locations, where they perform combinations of isolated dives and groups of dives (called “dive bouts”), suggesting a hierarchical and patchy distribution for their prey (Boyd, 1996; Mori, 1998). Studies on the diets of both species conducted in the North Sea show that self‐feeding and chick‐provisioning individuals capture mainly sandeel, sprat, young Atlantic herring, whiting, and cod (Anderson, Evans, Potts, Harris, & Wanless, 2014; Rindorf, Wanless, & Harris, 2000), selecting different prey sizes when self‐feeding and chick provisioning (Wilson et al. 2004). The two species differ in the number of prey carried back to the colony; razorbills typically bring several fish, while common guillemots feeding chicks bring back a single, usually large, fish (Thaxter et al., 2013). Stationary video cameras investigating underwater foraging behavior of common guillemots indicate high percentages of active foraging on individual prey and on low‐density shoals (Crook & Davoren, 2014). However, it is not currently well known how the foraging patterns of these marine predators are influenced by the density, distribution, and behavior of prey and how they adjust their behavior in response to changes in prey availability. Modeled foraging behavior for Peruvian booby (*Sula variegata*) and Guanay cormorant (*Phalacrocorax bougainvilliorum*) suggest that depth distribution is the primary factor for foraging success followed by abundance and then spatial configuration of prey (Boyd et al., 2016).

We used a combination of GPS and accelerometer data to explore the potential for these data to be used to assess I) prey availability in terms of the vertical distribution of prey encountered, and II) how individuals respond to differences in prey availability between contrasting visited food patches. We assume that seabird diving activities at a given location and depth are a function of both the diving capability of the species and the relative abundance, distribution, and type of prey throughout the water column. From the behavioral classification of accelerometer data, it is possible to detect pursuit or catching events (PCEs) occurring during dives, which are characterized by fast and sharp movements (Chimienti et al., 2016; Viviant et al., 2010). We further use the information obtained from PCE to explore prey availability and the profitability of food patches visited at two foraging levels: individual dives and dive bouts. We propose that I) the number of PCE in individual dives can be used as an indication of prey encountered through the water column, and II) the time spent pursuing and catching prey (PCT) in dive bouts can reveal different foraging strategies performed in response to changes in prey availability to the seabirds in the area visited.

## 2 MATERIALS AND METHODS

### 2.1 Data

Data were collected in 2014 and 2015 at two locations in Scotland (UK), Colonsay (56°3054N, 6°24021″W), and Fair Isle (59°22055″N, 1°48026″W). Axy‐Depth tags (TechnoSmArt, http://www.technos‐ mart.eu/), which comprise a tri‐axial accelerometer and a time–depth recorder (TDR), were deployed in combination with GPS tags (Gt‐120, IgotU) and mounted using Tesa tape (Tesa, Extra Power) on the backs of common guillemots and razorbills. GPS tags were set to record the location every 100 s, and accelerometers were set to record pressure (millibar, precision of 0.5 millibar) and temperature (°C, precision of 0.1°C) at 1 Hz and acceleration in three dimensions at 25 Hz (Chimienti et al., 2016). Data from four common guillemots and five razorbills were collected, respectively, from Colonsay (*n *= 2 guillemots in 2014) and Fair Isle (*n *= 5 razorbills in 2014 and *n *= 2 guillemots in 2015). For simplicity, the four guillemots will be referred to as COGU 1, COGU 2, COGU 3, and COGU 4. The five razorbills will be referred to as RAZO 1, RAZO 2, RAZO 3, RAZO 4, and RAZO 5.

Four razorbills (RAZO 1‐4) were tracked during incubation and one (RAZO 5) at the early‐stage of chick rearing (first week). COGU 1 and 2 (from Colonsay) were tracked during the chick‐rearing period, and the other two guillemots (COGU 3 and 4, from Fair Isle) were tracked while incubating. Capture and handling of birds were kept to a minimum (<5 min) and carried out under license from the British Trust for Ornithology. Devices were generally deployed on consecutive days; only COGU 3 and 4 were tracked at the same time. The devices were then recovered, when possible, as soon as the birds were back from a trip. The length of deployment varied from a minimum of a few hours (COGU 2 was back on the nest after a very short trip) to a maximum of five days. Mean body mass was 634.75 ± 32 g for razorbills and 878.75 ± 76 g for guillemots (Table S1). Sex and age were unknown. Animals can be affected by the attachment of devices during capture and handling (Mcmahon, Field, Bradshaw, White, & Hindell, 2008) to the psychological and physical stress of carrying a foreign body (Ropert‐Coudert, Knott, Chiaradia, & Kato, 2007; Wilson & Duffy, 1986). After being tracked, all animals successfully continued their breeding activities. No signs of impact arising from our GPS tracking were detected in these birds (Wakefield et al., 2017).

### 2.2 Data preparation

Data manipulation and behavioral pattern recognition of accelerometer data were conducted following the method developed in Chimienti et al. (2016), where an unsupervised learning algorithm *Expectation Maximization* was used to perform behavioral partitioning. A dive was defined as having a maximum depth of ≥1 m. By looking at the distribution of the interdive duration, a dive bout was defined as to be a group of dives in which the interdive durations were ≤300 s. Hence, a dive bout could be represented by an isolated dive or a group of dives.

In this study, we focus on PCE detected from the behavioral partitioning of data for each individual. During these events, animals performed fast and sharp movements in the water column, suggesting a type of activity that can be associated with pursuing and/or catching prey underwater (Chimienti et al., 2016). The number of PCE was defined as the number of times the algorithm detected pursuing and catching events across an entire whole dive. The number of PCE performed and time spent executing each event varied between dives. The total time spent for all PCE within a dive bout gave the PCT. For each dive, maximum dive depth and dive duration were also calculated. For each bout, we calculated the time spent underwater, defined as the sum of the dive durations within that bout. In total, 661 dives were recorded in 58 dive bouts for guillemots and 6214 dives in 156 dive bouts for razorbills (Table S1).

We extracted bathymetry data at a resolution of 1/8 arc minute (230 m) from the European Marine Observation and Data Network (EMODnet, http://www.emodnet-bathymetry.eu/). GPS positions were interpolated every 100 s using the R package adehabitatLT (Calenge 2006) to standardize the sampling interval and then matched with bathymetry and with dive based on the starting date and time for each dive. To distinguish between dives performed within the water column (“pelagic dives”) and dives performed to the sea floor (“benthic dives”), we applied a 10 m buffer to the bathymetric value at each dive location. Dives with a maximum depth within 10 m of the sea floor were considered to be benthic.

### 2.3 Individual dive models

In other diving marine predators (e.g., the little penguin (*Eudyptula minor*), Peruvian booby (*Sula variegata*), and Guanay cormorant (*Phalacrocorax bougainvilliorum*)), the distribution of prey capture events and dives in the water column match the local distribution of their prey (Boyd et al., 2016; Carroll et al., 2017). Therefore, we assumed that the number of PCE performed in each dive is a measure of foraging effort occurring in the presence of prey. We tested the hypothesis that the foraging effort is correlated to simple dive metrics such as maximum dive depth, duration, and type of dive performed (benthic vs. pelagic). Furthermore, we tested the interaction between dive depth and duration, hypothesizing that the value of one variable will depend on the value of the other. We aimed to highlight how the foraging strategies performed underwater (in terms of dive characteristics, e.g., depth, duration, and type) give an indication of prey encountered through the water column.

Due to the nonlinear relationships within the data, we fitted generalized additive mixed models (GAMM) for both species using the *gam* function in the mgcv package (Wood, 2006; see R code in S2). We tested different model structures considering both maximum dive depth and dive duration as main effects and/or as interactions within the same spline. Because dive depth and duration are on two different scales (space and time), we used anisotropic regression splines to model their interaction. We ran three different model structures: additive, additive plus interaction, and a model with only the interaction. We ran the models using maximum‐likelihood (ML) and selected the best structure according to AIC (Table S2). As individual dives were grouped within dive bouts, we ran the models with bout identity (bout ID) as a random effect. We assumed that bouts were serially independent of each other because they were distant in time. In all models, we assumed that the number of PCE followed a Poisson distribution, due to small sample size, animal ID was used as a fixed effect. The random effect was specified in the same way as the smoothers, as penalized regression term. Collinearity between the variables depth and duration was not an issue for these models (variance inflation factor <3). Following the guidelines of the *mgcv* package, the basis dimension of the penalized regression smoothers was set adequately small, see specific equations below. The residuals of all the models performed were checked for violations of model assumptions in terms of residual autocorrelation, heterogeneity, and normality. We performed the analysis using R version 3.3.1 (R Core Team 2016). The R code used for the models can be found in Appendix S2.

Razorbills only performed pelagic dives (see Results sections). For this reason, the models for razorbills included maximum dive depth and duration as explanatory variables (Equations (1), (2), (3), Table S2).

PCE∼Poisson(λ)

(1) $$\log\left(\lambda \right)\;=\;\alpha_{\mathrm{ID}}\;+\;f_{\mathrm{ID}}\left(\text{Duration}\right)\;+\;f_{\mathrm{ID}}\left(\text{Depth}\right)\;+\;\text{Animal ID}\;+\;\text{Bout ID}$$

(2) $$\log\left(\lambda \right)\;=\;\alpha_{\mathrm{ID}}\;+\;f_{\mathrm{ID}}\left(\text{Duration, Depth}\right)\;+\;\text{Animal ID}\;+\;\text{Bout ID}$$

(3) $$\begin{array}{cc} \log(\lambda )\;=\; & \alpha_{\mathrm{ID}}\;+\;f_{\mathrm{ID}}\left(\text{Duration}\right)+f_{\mathrm{ID}}\left(\text{Depth}\right)\\ & \;+\;f_{\mathrm{ID}}\left(\text{Duration, Depth}\right)\;+\;\text{Animal ID}\;+\;\text{Bout ID} \end{array}$$

where *PCE* is measured at the individual dive level; duration and depth are, respectively, the duration and depth of the dive; *f*_*ID*_(Duration) and *f*_*ID*_(Depth) are individual‐specific isotropic penalized cubic regression splines; and *f*_*ID*_(Duration, Depth) is an individual‐specific anisotropic bivariate penalized cubic regression spline. For all models, the basis dimension was set to 5. The use of individual‐specific splines allows for varying coefficient models according to each individual. Animal ID was set as a fixed effect and Bout ID as a random effect.

Given that the five razorbills were tracked from the same colony, in the same year, and performed the same foraging strategy, we also fitted the same relationship at a species level, by removing the interaction within the spline (Equations (3), [Link], (4), Table S2).

PCE∼Poisson(λ)

(4) $$\log\left(\lambda \right)\;=\;\alpha \;+\;f\left(\text{Duration}\right)\;+\;f\left(\text{Depth}\right)\;+\;\text{Animal ID}\;+\;\text{Bout ID}$$

(5) $$\log\left(\lambda \right)\;=\;\alpha \;+\;f\left(\text{Duration, Depth}\right)\;+\;\text{Animal ID}\;+\;\text{Bout ID}$$

(6) $$\begin{array}{cc} \log\left(\lambda \right)\;=\; & \alpha \;+\;f\left(\text{Duration}\right)\;+\;f\left(\text{Depth}\right)\\ & \;+\;f\left(\text{Duration, Depth}\right)\;+\;\text{Animal ID}\;+\;\text{Bout ID} \end{array}$$

where *f*(Duration) and *f*(Depth) are isotropic penalized cubic regression splines, and *f*(Duration, Depth) is an anisotropic bivariate penalized cubic regression spline. For all models, the basis dimension was set to 5, Animal ID was set as fixed effect, and Bout ID was set as random effect.

In contrast to the razorbills, the four guillemots performed both types of dives (pelagic and benthic, see Results section). The different areas used for foraging had different bathymetric profiles. As a consequence, the number of PCE was modeled for the two different types of dives considering maximum dive depth and duration, bathymetry, animal ID as fixed effects and Bout ID as a random effect (Equations (6), [Link], (7), Table S2).

PCE∼Poisson(λ)

(7) $$\begin{array}{cc} \log\left(\lambda \right)\;\;=\; & \;\alpha_{\mathrm{ClASS}}\;+\;f_{\mathrm{CLASS}}\left(\text{Duration}\right)\;+\;f_{\mathrm{CLASS}}\left(\text{Depth}\right)\\ & \;+\;\beta_{\mathrm{CLASS}}.\text{Bathymetry}+\text{Animal ID}\;+\;\text{Bout ID} \end{array}$$

(8) $$\begin{array}{cc} \log\left(\lambda \right)\;\;=\; & \alpha_{\mathrm{ClASS}}\;+\;\;f_{\mathrm{CLASS}}\left(\text{Duration, Depth}\right)\\ & \;+\;\beta_{\mathrm{CLASS}}.\text{Bathymetry}\;+\;\text{Animal ID}\;+\;\text{Bout ID} \end{array}$$

(9) $$\begin{array}{cc} \log\left(\lambda \right)\;=\; & \alpha_{\mathrm{ClASS}}\;+\;f_{\mathrm{CLASS}}\left(\text{Duration}\right)\;+\;f_{\mathrm{CLASS}}\left(\text{Depth}\right)\\ & \;+\;f_{\mathrm{CLASS}}\left(\text{Duration, Depth}\right)\;+\;\beta_{\mathrm{CLASS}}.\text{Bathymetry}\\ & \;+\;\text{Animal ID}\;+\;\text{Bout ID} \end{array}$$

where *f*_CLASS_(Duration) and *f*_CLASS_(Depth) are isotropic penalized cubic regression splines specific for each class of dive (benthic and pelagic), and *f*_CLASS_(Duration, Depth) is an anisotropic bivariate penalized cubic regression spline specific for each class of dive. The bathymetry was also considered in relation to the type of dive, as different foraging areas could have different bathymetric profiles. The basis dimensions for the isotropic and anisotropic splines were set to 3 and 5, respectively, and Animal ID was set as fixed effect and Bout ID as random effect.

### 2.4 Dive bout models

We tested the hypotheses that time spent underwater in a dive bout can predict PCT. We assumed that each dive bout was an indication of the animal sampling or exploiting a foraging patch. PCT was then assumed to indicate the effort invested within the whole foraging patch. We assumed that patch residence time (i.e., time spent underwater) was likely to increase with patch quality in heterogeneous habitats (Calcagno, Mailleret, Wajnberg, & Grognard, 2014; Wajnberg, Fauvergue, & Pons, 2000) giving insights into responses to different degrees of prey availability as perceived by the animals. Due to the nonlinear relationships in the data for both species, we modeled PCT as a function of the time spent underwater using GAMs for both species using the *gam* function from the *mgcv* package (Equations (9) and (10)). As our sample size was small, Animal ID was set as fixed effect in all models. The analysis was performed using the Tweedie distribution because time can only have positive values.

PCT∼Tweedie(μ,p)

(10) $$\log\left(\mu \right)\;=\;\alpha_{\mathrm{ID}}\;+\;f_{\mathrm{ID}}\left({\text{Time}}_{\mathrm{bout}}\right)\;+\;\text{Animal ID}$$

where p is 1.05, Time_bout_ is the time spent underwater in the bout, and *f*_*ID*_(Time_bout_) is the individual‐specific penalized cubic regression spline function of Time_bout_. The basis dimension was set to 3. This model (Equation (9)) fitted the guillemot data poorly, due mainly to overfitting and a nonlinear pattern in residuals. Therefore, both time spent catching and time spent underwater were log‐transformed, and a Gaussian model was then used for guillemots.

$$\log\left(\text{PCT}\right)∼N\left(\mu ,\sigma^{2}\right)$$

(11) $$\mu \;=\;\alpha_{\mathrm{ID}}\;+\;f_{\mathrm{ID}}\left(\log\left({\text{Time}}_{\mathrm{bout}}\right)\right)\;+\;\text{Animal ID}$$

where *f*_*ID*_(log(Time_bout_)) is the individual‐specific isotropic penalized cubic regression spline.

### 2.5 Comparison between the two species

To observe the general pattern among the two species and make a comparison, we considered individuals belonging to the same species as one group then looked at the ratio between the time spent pursuing and catching and time spent underwater. Both variables, time spent pursuing and catching and time spent underwater, were log‐transformed, and we fitted a linear model (LM) with the two species set as fixed effect (Equation (12)).

$$\log\left(\text{PCT}\right)∼\text{Gaussian}\left(\mu ,\sigma \right)$$

(12) $$\mu =\alpha +\log\left({\text{Time}}_{\mathrm{bout}}\right)+\text{Species ID}$$

## 3 RESULTS

### 3.1 Analysis of single dives

**Figure 2:**
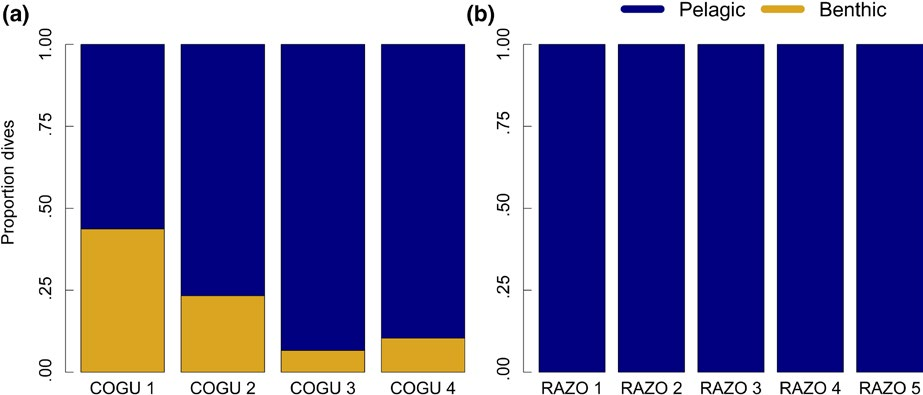
Proportion of dives classified as benthic and pelagic in four common guillemots (COGU 1–4) and five razorbills (RAZO 1–5)

Razorbills, tracked in 2014 from Fair Isle, performed only pelagic dives over a wide range of bathymetric profiles (Figures 2 and 7). Common guillemots showed variability in the proportion of benthic and pelagic dives performed in different locations (Figures 2 and 8). COGU 1 and COGU 2, tracked in 2014 from Colonsay, foraged in shallower areas compared to the other two individuals tracked from Fair Isle (Figure 8). COGU 1 and COGU 2 performed 44% and 23% of benthic dives, respectively. COGU 3 and COGU 4, tracked in 2015 from Fair Isle, performed 7% and 11% of benthic dives, respectively.

**Figure 3:**
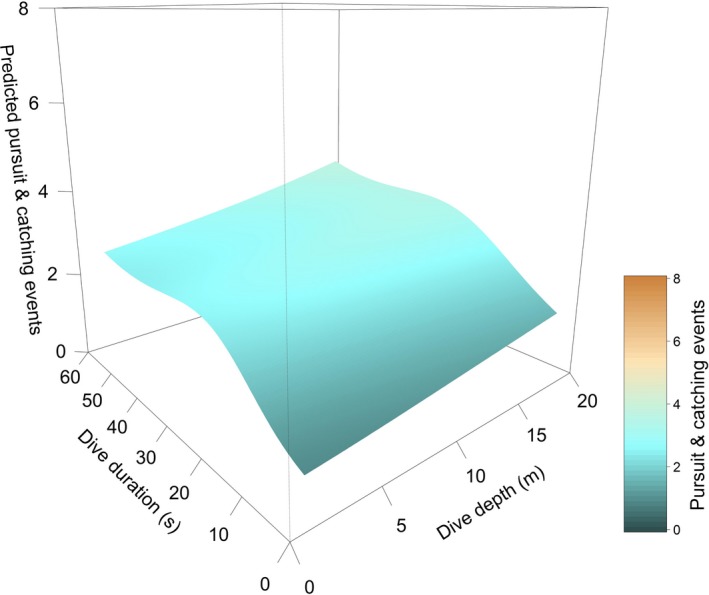
Overall prediction of the number of pursuit and catching events (PCEs) in relation to dive duration and depth in razorbills. The distribution of the data used for the model is in Figs. S2 and S3

For the models considering the individual ID for razorbills as an interaction within the spline (Equations (1), (2), (3)), the model considering both maximum dive depth and duration as main effects as well as their interaction within the same spline was the best fit (Equation (1), Table S2). For the models considering the behavior at species level (Equations (3), [Link], (4)), the model selection indicated that the model including only the interaction between maximum dive depth and duration was the best fit (Equation [Link], Table S2). Between the two resultant best models (Equations (1) and [Link]), the AIC showed a better fit for the model with a common spline for all individuals (Equation (1): R‐sq. (adj) = 0.26, AIC = 18262.75, Equation [Link]: R‐sq.(adj)  = 0.26, AIC = 18261.49, Table S2). The PCEs performed in each dive, as a response to the combination of dive duration and dive depth, generally increased with both dive time and depth. Generally, in all razorbills, the effect of the interaction between duration and depth was significant (Table S5) and the predicted PCEs peaked for dive duration between 20–40 s mainly in shallow dives (<10 m, Figure 3).

In the model considering different splines for each Animal ID (Equation (1)), the effect of dive duration was significant in all razorbills; dive depth was also significant in RAZO 2 and RAZO 3 and the interaction between the two variables was significant in all razorbills except RAZO 1 (Figs S2, S3 and S4, *p*‐value <.001, Table S4). For RAZO 1, RAZO 2, RAZO 4, and RAZO 5, the number of PCE increased with dive duration reaching a maximum between 2 and 4 for dive durations between 20 and 40 s (Fig. S4). Only in RAZO 1 did it subsequently decrease for longer dive durations. The response in the dive depth variable showed an increase in the number of PCE with depth. RAZO 3 showed a smaller increase in the number of PCE with dive duration, with no saturation/plateau as in the previous two individuals (Fig. S4). For RAZO 4, the highest number of PCE performed clustered in the first 10 m of the water column. For RAZO 5, the number of PCE remained stable as dive depth increased and the highest PCE were performed with longer and deeper dives (Fig. S4).

**Figure 4:**
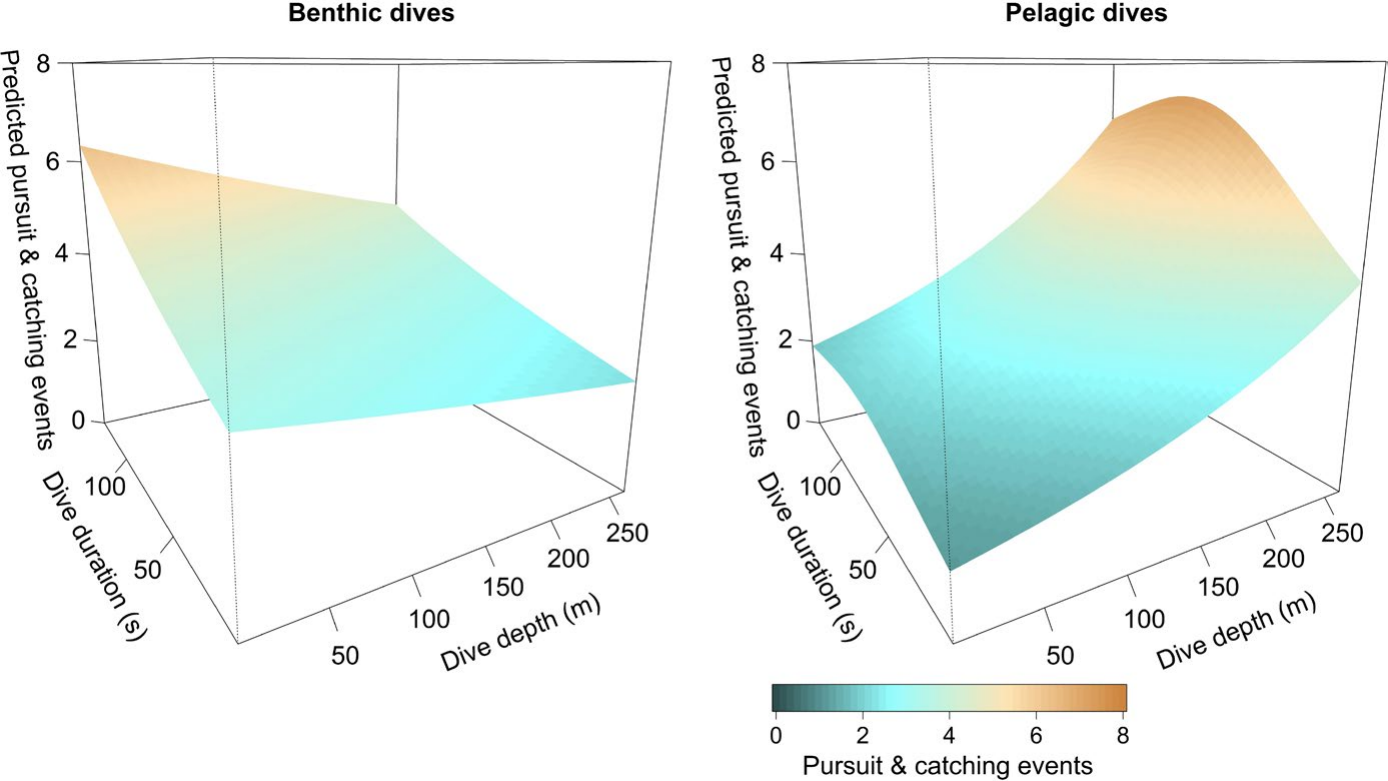
Prediction of the number of pursuit and catching events (PCEs) for benthic and pelagic dives given dive duration and depth in common guillemots. The distribution of the data used for the model is in Fig. S1

In guillemots, the model selection indicated equal support for the model including maximum dive depth and duration as main effects and for the model including depth, duration, and their interaction (Table S2). We therefore selected the model with the simpler structure (Equation (6)). The analysis of the number of PCE performed in each dive in response to dive duration and dive depth resulted in different predictions for benthic and pelagic dives. The effect of both dive duration and depth was significant for pelagic dives, while for benthic dives, only the effect of dive duration was significant (Figure 4, Fig. S1, Table S3). During benthic dives, the predicted PCE performed for each dive slightly declined with dive depth and duration. During pelagic dives, the predicted PCE increased with both dive duration and depth, increasing from a minimum value of about 1.5 for shallow and short dives (<50 m and <50 s) to over 4 in deep and long dives (dives >200 m and >100 s) (Figure 4, Fig. S1).

### 3.2 Analysis of dive bouts

**Figure 5:**
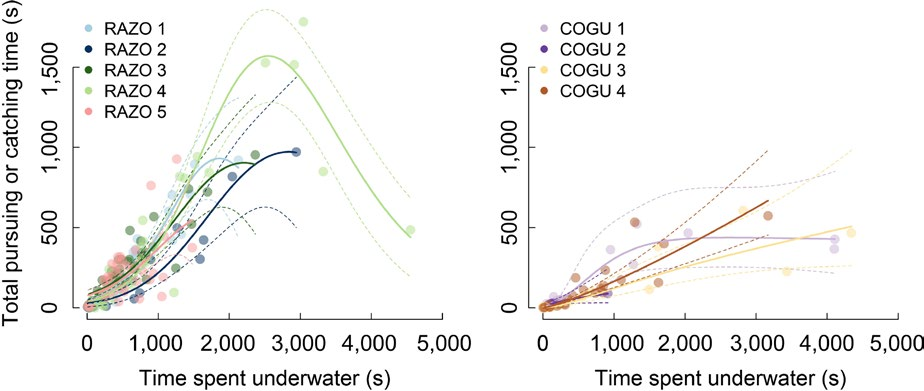
Total time spent pursuing and catching (PCT) predicted by the time spent underwater during a foraging bout for individual razorbills (RAZO 1, RAZO 2, RAZO 3, RAZO 4 and RAZO 5, left panel) and common guillemots (COGU 1, COGU 2, COGU 3, COGU 4, right panel) Continuous lines indicate model prediction and dashed lines ±95% confidence intervals

**Figure 6:**
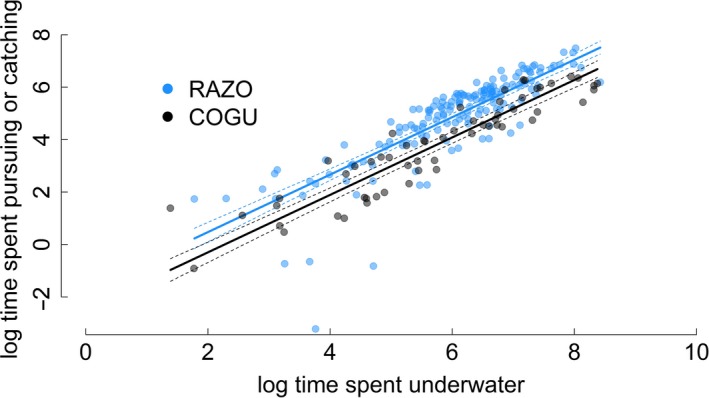
Total time spent pursuing and catching predicted by the time spent underwater during a foraging bout for all common guillemots and razorbills on log scale (ln). Continuous lines indicate model prediction and dashed lines ±95% confidence intervals

The analysis of the total PCT as a function of the time spent underwater in each dive bout showed different patterns between the two species and fairly consistent results among individuals of the same species, especially in razorbills (Figure 5, Table S6 and S7). The five razorbills showed a rapid increase in the total PCT as the time spent in the bout increased up to 1500 s. For longer dive bouts (max time spent underwater = 4550 s), the PCT started to reach an asymptote. For three of the four guillemots, the PCT in a dive bout linearly increased with the total time spent underwater. One guillemot, COGU 1, showed a different relationship where PCT reached an asymptote for dive bouts longer than 1000 s. The analysis of the comparison of PCT as a function of the time spent underwater between the two species (Figure 6) showed the ratio between bout time and PCT more clearly. The difference between the species was significant (*p*‐value <.001, Table S8). The ratio between PCT and time spent underwater was higher in razorbills than guillemots, indicating that razorbills spend relatively more time pursuing and catching than guillemots.

## 4 DISCUSSION

While foraging, predators may use hierarchical foraging tactics, responding to patches at a variety of spatial and temporal scales, to maximize their chances of encountering prey aggregations (Fauchald et al., 2000; Fryxell et al., 2008; Weimerskirch et al., 2005). In the marine environment, both predators and prey can be highly mobile and difficult to monitor simultaneously. When foraging, seabirds typically perform hierarchical movement patterns performing “area‐restricted search” (ARS) movements or series of dives in each foraging location, reflecting the spatial and temporal dynamics of food patches (Fauchald et al., 2000; Regular et al., 2013; Weimerskirch et al., 2005). As marine top predators, seabirds have been shown to provide unique insights on the status and changes of marine ecosystems (Piatt, Sydeman, & Wiese, 2007).

In this study, we demonstrate the use of high‐frequency foraging movement data to explore the feeding strategy of two species of diving seabirds in more detail. We propose using the number of PCE as an indicator of the effort that an animal chooses to invest in a specific location (Thums, Bradshaw, Sumner, Horsburgh, & Hindell, 2013; Watanabe et al., 2014). By exploring the number of PCE in relation to dive depth, duration, and type of dive performed (benthic vs. pelagic), we highlight sections of the water column in which animals forage. We then explore the use of PCT in response to time spent underwater within a dive bout to highlight patch level foraging processes.

### 4.1 PCE as indicator for prey encounters in the water column

The depth distribution of prey plays an important role in how predators use their habitat (Benoit‐Bird et al., 2013; Boyd et al., 2016; Carroll et al., 2017). By exploring the relationship between the number of PCE with dive depth and duration, we have shown that two species, guillemots and razorbills, clearly made different decisions while exploiting the water column. We infer that these different behaviors are driven by differing prey availability.

Although razorbills are capable of diving deeper to observed diving depths beyond 35 m (Dall'Antonia, Gudmundsson, & Benvenuti, 2001; Thaxter et al., 2010), the razorbills in our sample never performed benthic dives and consistently foraged only in the top 15–20 m of the water column (Figures 2, 3 and Fig. S2). Within each dive, the depths at which the higher number of PCE was performed were variable, suggesting that prey were available throughout the upper 20 m of the water column. However, the time during the dive (between 20 and 40 s) when PCEs were highest was more predictable across individuals. This timing, and analysis of dives in previous work (Chimienti et al., 2016), suggests that razorbills perform their catching events on their way back up through the water column and are therefore targeting habitats where prey are available in the upper surface waters.

**Figure 7:**
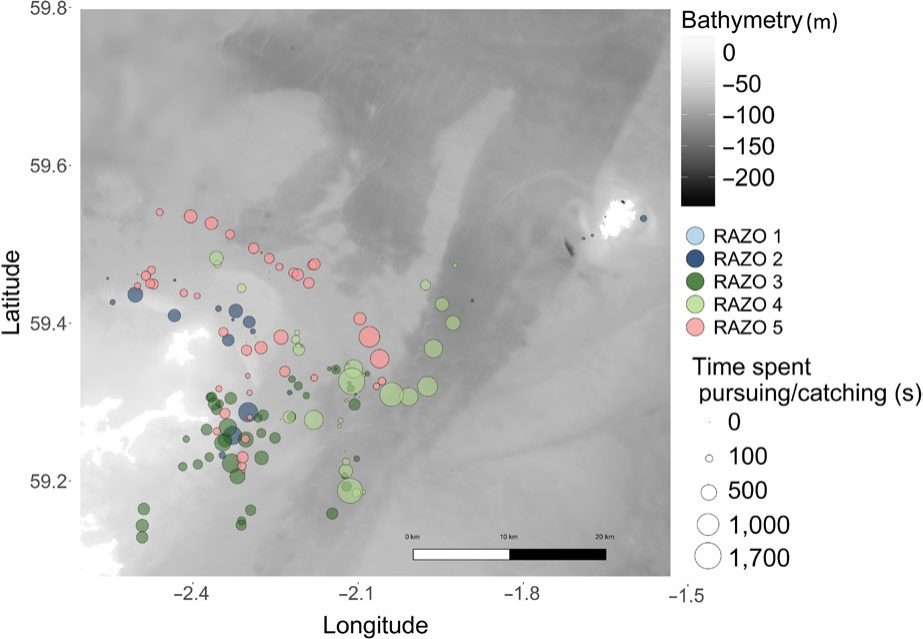
Location of foraging bouts of the five razorbills tracked on Fair Isle. Bathymetry shown in gray and land in white. The size of each bout location represents time spent pursuing and catching (PCT)

As shown in other species of diving seabirds, such as Peruvian booby, Guanay cormorant, and little penguin, the probability of preforming foraging dives was shown to be predicted by the vertical distribution of prey (Benoit‐Bird, Kuletz, Heppell, Jones, & Hoover, 2011; Benoit‐Bird et al., 2013; Boyd et al., 2016; Carroll et al., 2017). Strong tidal currents and upwelling currents move prey toward the water surface and therefore increase their catchability (Embling, Sharples, Armstrong, Palmer, & Scott, 2013; Enstipp, Grémillet, & Jones, 2007; Stevick et al., 2008). Despite greater physiological capabilities (Thaxter et al., 2010), razorbills performed short and shallow dives in areas characterized by wide bathymetric variation, possibly indicating foraging decisions driven by profitability of the food patches (Figure 7).

**Figure 8:**
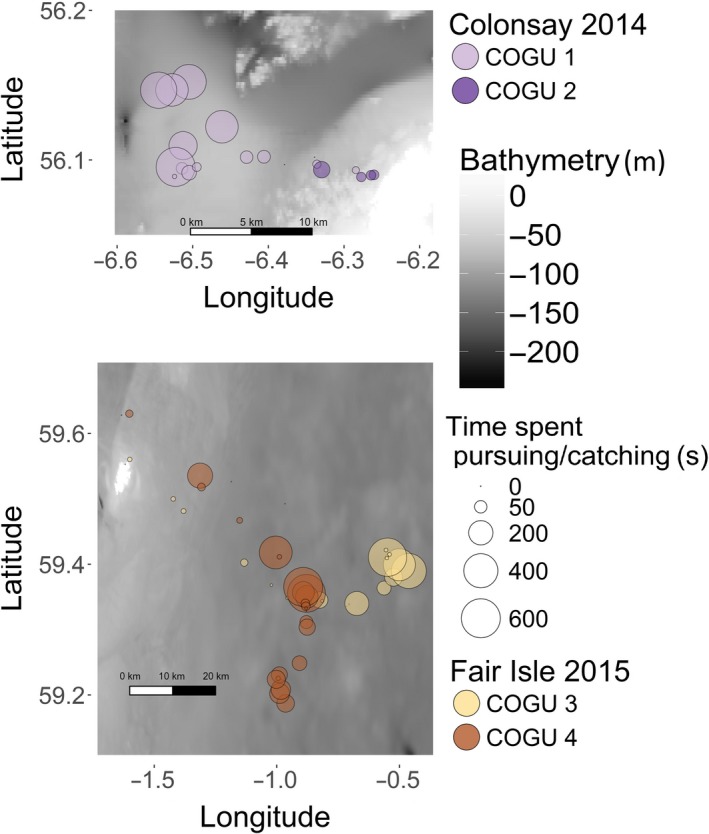
Location of foraging bouts of the two guillemots tracked on Colonsay (top panel) and the two guillemots tracked on Fair Isle (bottom panel). Bathymetry shown in gray and land in white. The size of each bout location represents time spent pursuing and catching (PCT)

However, physical characteristics might not always be exploited by common guillemots when foraging (Benoit‐Bird et al., 2013). Guillemots sampled deep parts of the water column and the sea floor (Figures 2 and 4) at both locations in which they were tagged (Figure 8). The area used around Colonsay was much shallower than the area used around Fair Isle. Different environmental conditions (e.g., type of prey encountered through the water column) and bathymetric profiles around the colonies can have an impact on type of behavior performed (Figures 8 and S1). Despite already feeding in the relatively deep waters around Fair Isle, both COGU 3 and COGU 4 also opted for benthic dives in this region, performing the deepest dives ever recorded for the species, reaching 250 m (max depth previously recorded 177 m (Regular, Hedd, & Montevecchi, 2011)).

By switching between pelagic and benthic prey, and performing different PCE between pelagic and benthic locations, diving marine predators adjust their behavior to maximize the opportunities presented in the range of trade‐offs between pelagic and benthic prey availability (Benoit‐Bird et al., 2011; Thums et al., 2013). Guillemots are known to exploit a broad range of fish and invertebrate prey (Anderson et al., 2014; Elliott et al., 2008), and it has been shown that the effect of the pressure throughout the water column and type of prey caught can affect the type of movement performed as well as the number of PCE (Cook, Kato, Tanaka, Ropert‐Coudert, & Bost, 2010; Elliott et al., 2008).

Guillemots’ diving capability coupled with varying energetic content of different types of prey affects the level of energy used to perform PCE. Within a dive, the majority of PCEs were usually performed after the searching phase (bottom of a dive), but, occasionally, the PCEs were also performed during the ascending phase (Chimienti et al., 2016). The number of benthic PCEs slightly increased with dive time and slightly decreased with dive depth. The foraging areas used around Colonsay were shallower than those used around Fair Isle (Figure 8). At shallower depths (e.g., 50 m), guillemots could spend more time searching/catching than at deeper depths (e.g., >150 m) resulting in a slightly higher number of PCE in shallow waters than in deep waters and showing the effect of the greater effort required to reach deep patches.

When performing pelagic dives, the type of prey found in deeper waters may be more worthwhile than the prey found during short or shallow pelagic dives. When pelagic dives are deeper, PCEs peak, indicating prey aggregations found while exploiting the deeper parts of the water column. Type of prey caught and brought back to the chicks during the breeding season can be also affected by atmospheric conditions. Under high wind conditions, guillemots switched from feeding their offspring on schooling fish, to preying on amphipods caught while performing benthic dives (Elliott et al., 2014). It is not known if by switching type of dive, and possibly the type of prey pursued, a different amount of effort is required.

### 4.2 Species‐specific foraging strategies: responses to changes in prey availability

Animal behavior and ecology are intricately linked to environmental conditions which are dynamic in space and time (Phillips, Croxall, Silk, & Briggs, 2008; Shaffer et al., 2006; Weimerskirch et al., 2005). Seabirds perform behaviors over multiple spatiotemporal scales (e.g., dives nested within dive bouts), and clear associations are recorded corresponding to biophysical phenomena that lead to patchiness (Cox, Scott, & Camphuysen, 2013; Pinaud, 2007; Pinaud, Cherel, & Weimerskirch, 2005; Waggitt et al., 2016).

Within‐patch movements in foraging individuals have been explained by the change in movement parameters based on their experience of resource encounters. Earlier studies have defined and observed ARS movements in animals that reduce movement speed and/or increase sinuosity in response to a highly clumped resource distribution (Bailleul, Lesage, & Hammill, 2010; Barraquand & Benhamou, 2008; Fauchald et al., 2000). By exploring the relationship between PCT and total time spent underwater in each foraging bout, we highlight the intensity of within‐patch pursuing and catching movements across the two species and give insights on patch profitability as perceived by the animals.

PCT within dives or bouts should not be taken as a direct measure of the number of prey caught. PCEs are not always successful and, depending on the type of prey targeted and the type of dive performed (pelagic or benthic), animals move differently, investing different effort, which is translated into different numbers of catching attempts recorded. Studies conducted under natural conditions and including validation datasets have used both bird‐borne video cameras (Ponganis et al., 2000; Takahashi et al., 2004; Watanabe & Takahashi, 2013; Watanuki et al., 2008) and stationary underwater video cameras (Crook & Davoren, 2014) to observe the animal`s foraging behavior and the type of prey caught. Data collected from Adélie penguins highlighted the possibility of both diminishing return, increasing return, and constant return gain functions (Watanabe et al., 2014). The majority of the gain functions between pursuit and catching events and time spent underwater showed a sigmoid curve, supporting the assumption that the animals were feeding on large prey aggregations that were being depleted or dispersed over time (Watanabe et al., 2014).

As observed in penguins (Watanabe et al., 2014), razorbills showed a similar diminishing shape in the gain function, calculated as PCT while foraging in a patch (dive bout) (Figure 5). About 94% of the observations for the five razorbills in this study fell within dive bouts of duration <2000 s. The relationship reaches an asymptote, possibly suggesting a physiological limit in the amount of effort that an individual can spend within a bout. The decrease observed in one individual is driven by two data points and should not be taken as a meaningful feature of the model. Observations of dive bouts longer than 2000 s were rare and also had a correspondingly high variability in PCT.

According to the marginal value theorem (MVT), if patches vary in quality (profitability), a predator should leave the patch when the marginal capture rate falls to the average rate for the habitat (Charnov, 1976). As the animal forages in the patch, the availability of food in the patch diminishes. As a consequence, the instantaneous rate of food gain drops. The forager's expectation of the profitability of a patch can be influenced by the experience of previously visited patches (Vásquez, Grossi, & Marquez, 2006). In poor patches, where capture is rarer, predators might take longer to assess the local profitability than in rich patches where prey were frequently encountered, with consequent overuse of poor patches (Esposito, Incerti, Giannino, Russo, & Mazzoleni, 2010). The overuse of poor patches can lead to a low expectation of environmental profitability, with consequent overuse of all patches (Esposito et al., 2010). Razorbills often performed short dive bouts of <1000 s duration (Figures 5, 6, 7), which could represent very good foraging patches. The sigmoid effect can perhaps be an indication of spatial structures of prey swarms, diminishing food, satiety, or tiredness affecting the total time spent pursuing and catching, as well as the effect of less profitable patches after highly profitable ones (Watanabe et al., 2014; Watkins & Murray, 1998).

Common guillemots exhibited longer and deeper dives than razorbills, performed fewer dives and dive bouts, and performed types of searching behaviors underwater that were not observed in razorbills (Chimienti et al., 2016). PCT is associated with events during which the animals might effectively attempt to catch prey. The differences in the relationship between time spent underwater and PCT between the two species, as well as the distribution of the data points around the prediction, clearly indicate different foraging choices (Figure 6).

At comparable time spent in a patch for the two species, the time spent pursuing and catching was much lower in guillemots. By reaching deeper patches than razorbills, guillemots travel further underwater, probably reducing time spent in PCE. However, the cost of moving through the water column changes with depth (Lovvorn, Liggins, Borstad, Calisal, & Mikkelsen, 2001) and the type of prey targeted can also affect the time budget within a dive. Precise information on energetic expenditure of each dive and type of prey caught can disentangle the effect of targeting different patches on a cost/benefit functional response.

Fish schools close to the sea floor can be larger and less dense during neap tides compared to shallow pelagic fish schools (Embling et al., 2013). Feeding on deep dispersed pelagic or benthic prey and targeting small prey patches or isolated prey might require a different foraging strategy than that employed for schooling fish (Crook & Davoren, 2014; Thums et al., 2013). We propose that the observed species‐specific foraging strategies are the result of species‐specific optimal foraging decisions taken according to perceived availability and profitability of foraging patches encountered as a response to dynamic changes in both prey availability and characteristics of heterogeneous environments.

In order to disentangle effects of prey encounter rate on dive time, further effort should be invested in validating these new insights with data on prey availability and distribution and in building context‐dependent dynamic models (Morales et al., 2010). Studies combining tracking data with prey survey data are very rare (Boyd et al., 2015; Carroll et al., 2017). Research on foraging site selection and how selection patterns adapt in response to changes in prey availability are fundamental for understanding the scale at which predators relate to their prey and invest more effort. Furthermore, knowledge of how and where predators select and exploit food patches would improve the design of conservation measures and the planning of marine habitat use.

### 4.3 The future of combined movement data

Tracking devices have been used previously as a method for quantifying important areas used by wild animals (Block et al., 2011; Hussey et al., 2015; Kays et al., 2015). Despite their potential, tracking studies often provide data on only a few individuals, depending on species studied and type of device used, and are often carried out in a small number of years because of the cost of the devices or other logistical constraints. In our study, the small sample size obtained does not allow us to estimate the degree of variation in the behaviors observed between individuals belonging to the same colony, across colonies or years. However, we can combine data collected by traditional tracking devices with data collected by more recent tracking technology, recording orientation, acceleration, temperature, and environmental characteristics (Richard, Cox, Picard, Vacquié‐Garcia, & Guinet, 2016). This combination allows researchers to infer information about habitat selection and animal decision making across types of data and individuals.

Recently, a few studies started to build predictive models considering information on pursuit and catching events detected by accelerometers using dive data only to assess foraging success and test optimal foraging theory (Foo et al., 2016; Jouma'a et al., 2015; Viviant, Monestiez, & Guinet, 2014). Dive duration and depth are generally good predictors of PCE. We further emphasize the need to include additional types of dive metrics when inferring foraging success from dive data only (such as dive type or shape), especially in species that use the water column to search for and catch prey.

Further research should also be directed toward building models for transferring information across multiple spatiotemporal scales and based on behavioral information acquired from different tracking devices. Examining behavior at different temporal and spatial scales has the potential to reveal animal movement decisions and reasons for observed changes in foraging patterns. Ultimately, combining multiscale modeling approaches with behavioral information can provide an opportunity to progress from the movement ecology of a few individuals to descriptions of population‐level habitat use.

## 5 CONCLUSIONS

Understanding how marine predators select and exploit different types of prey patches from high‐frequency movement data offers the unique opportunity to comprehend the behavioral ecology behind different movement patterns and improves our understanding of how animals might respond to changes in prey distributions. By looking at the foraging behavior of two species of seabirds, we have gained new insights into the different strategies used when pursuing prey throughout the water column. We propose that the information on pursuit and catching events can be used as a proxy for perceived prey availability throughout the water column. The variation in time spent pursuing and catching across dive bouts provided information on the behavioral responses to different levels of prey availability. Razorbills exploited areas with high variation in bathymetry and performed only pelagic dives, most likely exploiting fish aggregations distributed at shallow depths, as indicated by the distribution of the pursuit and catching events. In contrast, guillemots were more flexible in their behavior, switching between benthic and pelagic dives, and had rates of pursuit and catching events indicating that they were probably targeting different prey aggregations than razorbills.

The analysis performed in this study depended on data collected at very fine spatiotemporal scales and was performed on few individuals. Including such detailed information in movement models looking at broader scales will provide solid foundations for the analysis of long‐term movement datasets. These new modeling approaches, in conjunction with fine‐scale data about prey density and distributions, will play an important role in clarifying the type of habitat and prey selected as well as effort invested by predators in specific areas. Understanding why, where, and how these animals use their habitat has the potential to inform species‐specific survey plans and has a direct impact when determining the effects of anthropogenic developments and changing environments on foraging behavior.

## DATA AVAILABILITY

Data available from the Dryad Digital Repository: https://doi.org/10.5061/dryad.f0780

## COMPETING INTERESTS

We confirm that there are no known conflicts of interest associated with this publication.

## AUTHOR`S CONTRIBUTIONS

MC conceived and designed the study, collected the data, designed the methodology, analyzed the data, prepared figures and/or tables, led the writing of the manuscript. TC contributed to designing the methodology and interpretation of the data. EO and MB conceived and designed the study, lead the project for the data collection, and contributed to the interpretation of the data. ID, JMJT, and BES conceived and designed the study and contributed to the interpretation of the data. All authors contributed critically to the drafts and gave final approval for publication.
