# Supplementary material for: Taking movement data to new depths: Inferring prey availability and patch profitability from seabird foraging behavior
Source: Ecol Evol. 2017 Oct 25;7(23):10252–65. doi: 10.1002/ece3.3551 (PMC5723613; doi:10.1002/ece3.3551)
Supplement: Supplementary file 1 [file ECE3-7-10252-s001.docx]

**Table S1:** Date of deployment and recovery, breeding success, breeding status, colony ID, body mass (g) and number of dives and dive bout included in the analyses for the 5 razorbills and 4 guillemots. NR = weight taken at the colony but not reported. B/1L = chick fledged

| Animal ID | Deployment | Recovery | Breeding success | Breeding status | Colony | Mass (g) | N Dives | N Bouts |
| --- | --- | --- | --- | --- | --- | --- | --- | --- |
| RAZO 1 | 12/05/2014 | 15/05/2014 | B/1 L | incubating | Fair Isle | NR* | 1040 | 21 |
| RAZO 2 | 24/05/2014 | 27/05/2014 | Unknown | incubating | Fair Isle | 614 | 599 | 17 |
| RAZO 3 | 14/05/2014 | 19/05/2014 | B/1 L | incubating | Fair Isle | 640 | 1691 | 45 |
| RAZO 4 | 11/06/2014 | 15/06/2014 | B/1 L | incubating | Fair Isle | 670 | 1834 | 31 |
| RAZO 5 | 11/06/2014 | 15/06/2014 | B/1 L | chick rearing | Fair Isle | 615 | 1050 | 42 |
| COGU 1 | 17/06/2014 | 19/06/2014 | B/1 L | chick rearing | Colonsay | 850 | 199 | 14 |
| COGU 2 | 27//06/2014 | 27/06/2014 | B/1 L | chick rearing | Colonsay | 785 | 26 | 4 |
| COGU 3 | 26/06/2015 | 29/05/2015 | Unknown | incubating | Fair Isle | 930 | 239 | 17 |
| COGU 4 | 26/06/2015 | 29/05/2015 | Unknown | incubating | Fair Isle | 950 | 197 | 23 |

.

**Table S2**: model comparison performed on three different model structures considering both maximum dive depth and dive duration as main effects and/or as interaction within the same spline.

| Species | Model structure | Residual Deviance | AIC | R-sq (adj) |
| --- | --- | --- | --- | --- |
| Razorbills  (Animal ID as interaction) | f_ID_(Duration) + f_ID_(Depth) + f(Bout_N, bs = "re") + Animal_ID | 2955.5 | 18266.87 | 0.26 |
|  | f_ID_(Duration, Depth) + f(Bout_N, bs = "re") + Animal_ID | 2959.0 | 18265.44 | 0.26 |
|  | f_ID_(Duration) + f_ID_(Depth) + f_ID_(Duration, Depth) + f(Bout_N, bs = "re") + Animal_ID | 2954.6 | 18262.75 | 0.26 |
| Razorbills  (species model) | f(Duration) + f(Depth) + f(Bout_N, bs = "re") + Animal_ID | 2979.8 | 18263.12 | 0.26 |
|  | f(Duration, Depth) + f(Bout_N, bs = "re") + Animal_ID | 2983.9 | 18261.49 | 0.26 |
|  | f(Duration) + f(Depth) + f(Duration, Depth) + f(Bout_N, bs = "re") + Animal_ID | 2980.4 | 18264.96 | 0.26 |
|  | | | |  |
| Common Guillemots | f_CLASS_(Duration) + f_CLASS_(Depth) + f(Bout_N, bs = "re") + β_CLASS_.Bathymetry+ Animal_ID | 614.89 | 2557.667 | 0.3 |
|  | f_CLASS_(Duration, Depth) + f(Bout_N, bs = "re") + β_CLASS_.Bathymetry+ Animal_ID | 620.97 | 2561.043 | 0.3 |
|  | f_CLASS_(Duration) + f_CLASS_(Depth) + f_CLASS_(Duration, Depth) + f(Bout_N, bs = "re") + β_CLASS_.Bathymetry+ Animal_ID | 614.89 | 2557.666 | 0.3 |

**Table S3**: Approximate significance of smooth terms of the model performed on individual dives for guillemots (Eq 7 main text).

| Smooth terms | edf | p-value |
| --- | --- | --- |
| s(Duration):Pelagic | 1.812 | <0.05 |
| s(Duration):Benthic | 1.000 | <0.05 |
|  |  |  |
| s(Depth): Pelagic | 1.000 | <0.01 |
| s(Depth): Benthic | 1.000 | >0.05 |
|  |  |  |
| s(Bout N, bs=”re”) | 24.099 | <0.001 |

**Table S4:** Approximate significance of smooth terms of the model performed on individual dives for razorbills (Eq 3 main text).

| Smooth terms | edf | p-value |
| --- | --- | --- |
| s(Duration):Animal ID1 | 2.90873 | <0.001 |
| s(Duration):Animal ID2 | 1.00014 | <0.01 |
| s(Duration):Animal ID3 | 1.00015 | <0.001 |
| s(Duration):Animal ID4 | 1.00002 | <0.001 |
| s(Duration):Animal ID5 | 1.00014 | <0.001 |
|  |  |  |
| s(Depth):Animal ID1 | 1.00020 | >0.05 |
| s(Depth):Animal ID2 | 1.00006 | <0.01 |
| s(Depth):Animal ID3 | 1.00004 | <0.001 |
| s(Depth):Animal ID4 | 2.07448 | >0.05 |
| s(Depth):Animal ID5 | 1.00010 | >0.05 |
|  |  |  |
| ti(Duration,Depth): Animal ID1 | 0.01231 | >0.05 |
| ti(Duration,Depth): Animal ID2 | 1.61063 | <0.001 |
| ti(Duration,Depth): Animal ID3 | 1.44528 | <0.001 |
| ti(Duration,Depth): Animal ID4 | 1.91730 | <0.001 |
| ti(Duration,Depth): Animal ID5 | 1.87717 | <0.001 |
|  |  |  |
| s(Bout N, bs=”re”) | 26.79436 | <0.001 |

**Table S5:** Approximate significance of smooth terms of the model performed on individual dives for razorbills (Eq 5 main text).

| Smooth terms | edf | p-value |
| --- | --- | --- |
| te(Duration,Depth) | 3.973 | <0.001 |
| s(Bout N, bs=”re”) | 29.577 | <0.001 |

**Table S6:** parametric coefficients estimates of the model performed on dive bouts for razorbills (Eq 10 main text).

| Parametric coefficients | Estimate | Std Error | p-value |
| --- | --- | --- | --- |
| (Intercept) | 5.18046 | 0.17121 | <0.001 |
| RAZO 2 | -0.56744 | 0.28026 | 0.0488 |
| RAZO 3 | 0.20386 | 0.19159 | >0.05 |
| RAZO 4 | -0.07345 | 0.21447 | >0.05 |
| RAZO 5 | 0.05269 | 0.25641 | >0.05 |

**Table S7:** parametric coefficients estimates of the model performed on dive bouts for guillemots (Eq 11 main text).

| Parametric coefficients | Estimate | Std Error | p-value |
| --- | --- | --- | --- |
| (Intercept) | 4.3577 | 0.2798 | <0.001 |
| COGU 2 | -0.5502 | 0.3983 | >0.05 |
| COGU 3 | -0.9628 | 0.3141 | <0.05 |
| COGU 4 | -0.7111 | 0.3004 | <0.05 |

**Table S8:** parametric coefficients estimates of the model performed for the comparison between the two species (Eq 12 main text).

| Parametric coefficients | Estimate | Std Error | p-value |
| --- | --- | --- | --- |
| (Intercept) | -2.48829 | 0.27434 | <0.001 |
| Time spent underwater | 1.09571 | 0.04382 | <0.001 |
| Razorbill | 0.77189 | 0.13307 | <0.001 |


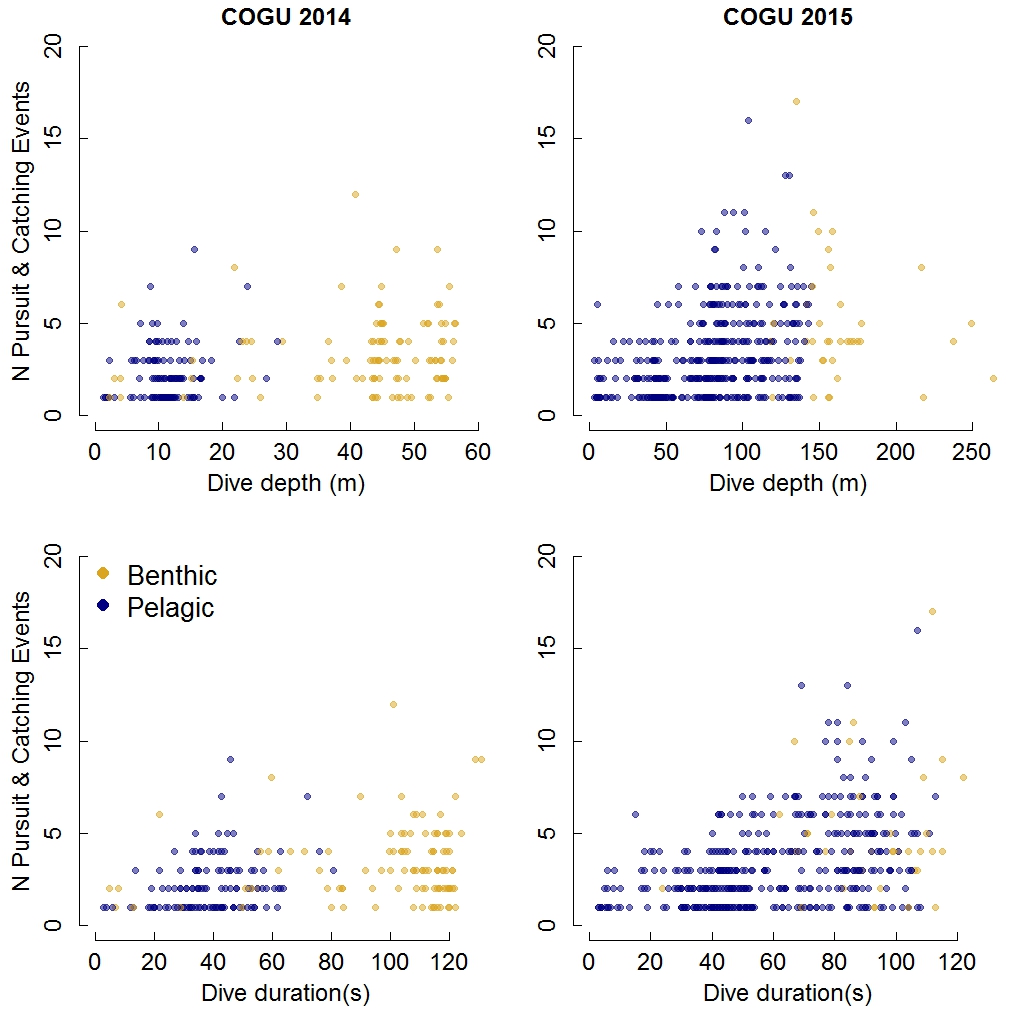


Figure S1: Distribution of number of pursuit & catching events (PCE) against dive depth and dive time in guillemots from Colonsay (left column, COGU 2014) and in common guillemots from Fair Isle (right column, COGU 2015).


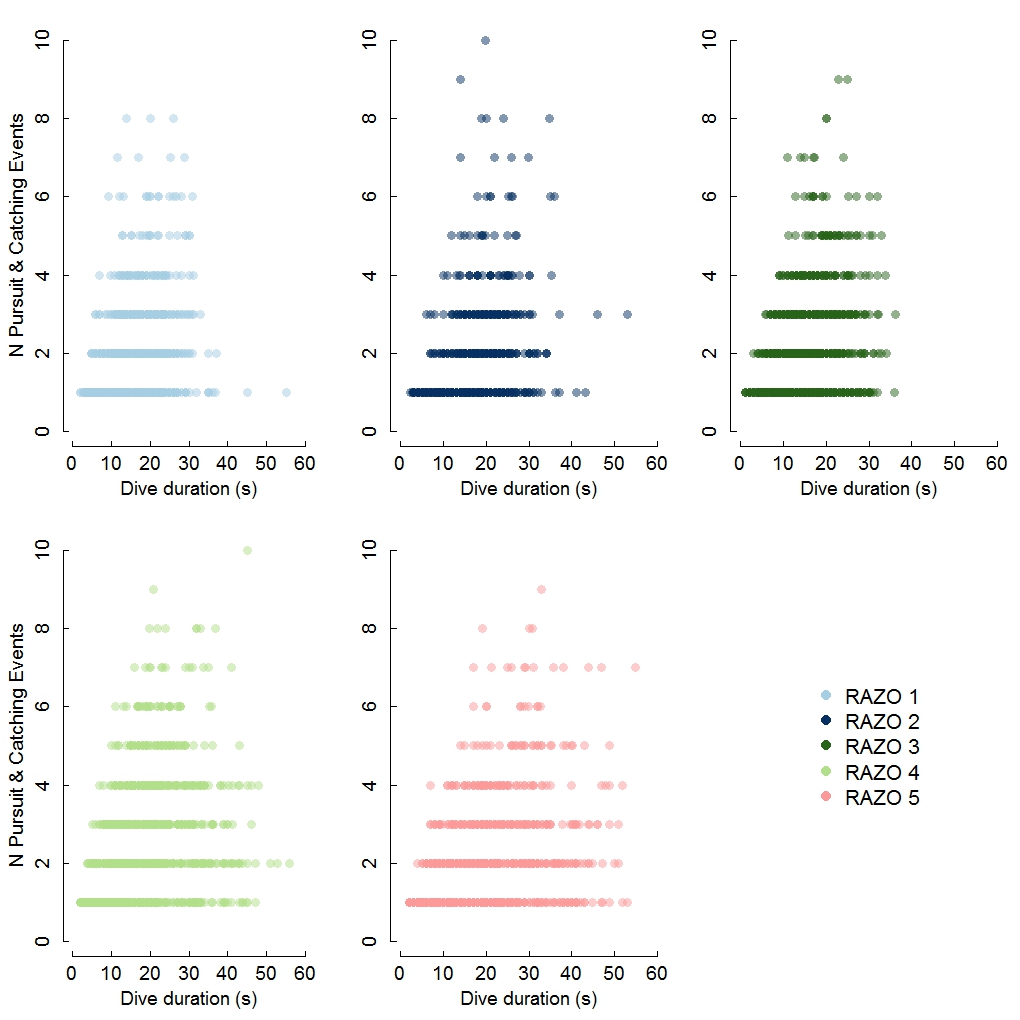


Figure S2: Distribution of number of pursuit & catching events (PCE) against dive duration in razorbills.


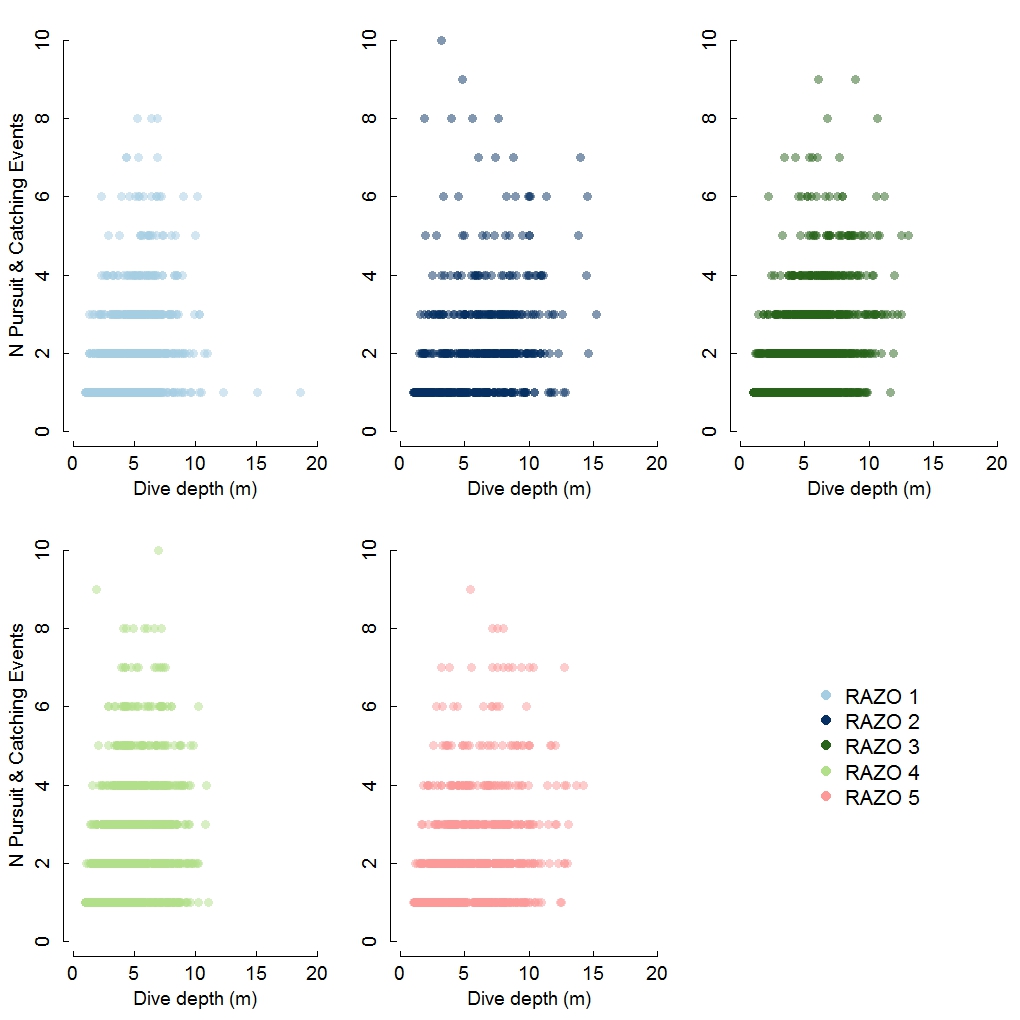


Figure S3: Distribution of number of pursuit & catching events (PCE) against dive depth in razorbill


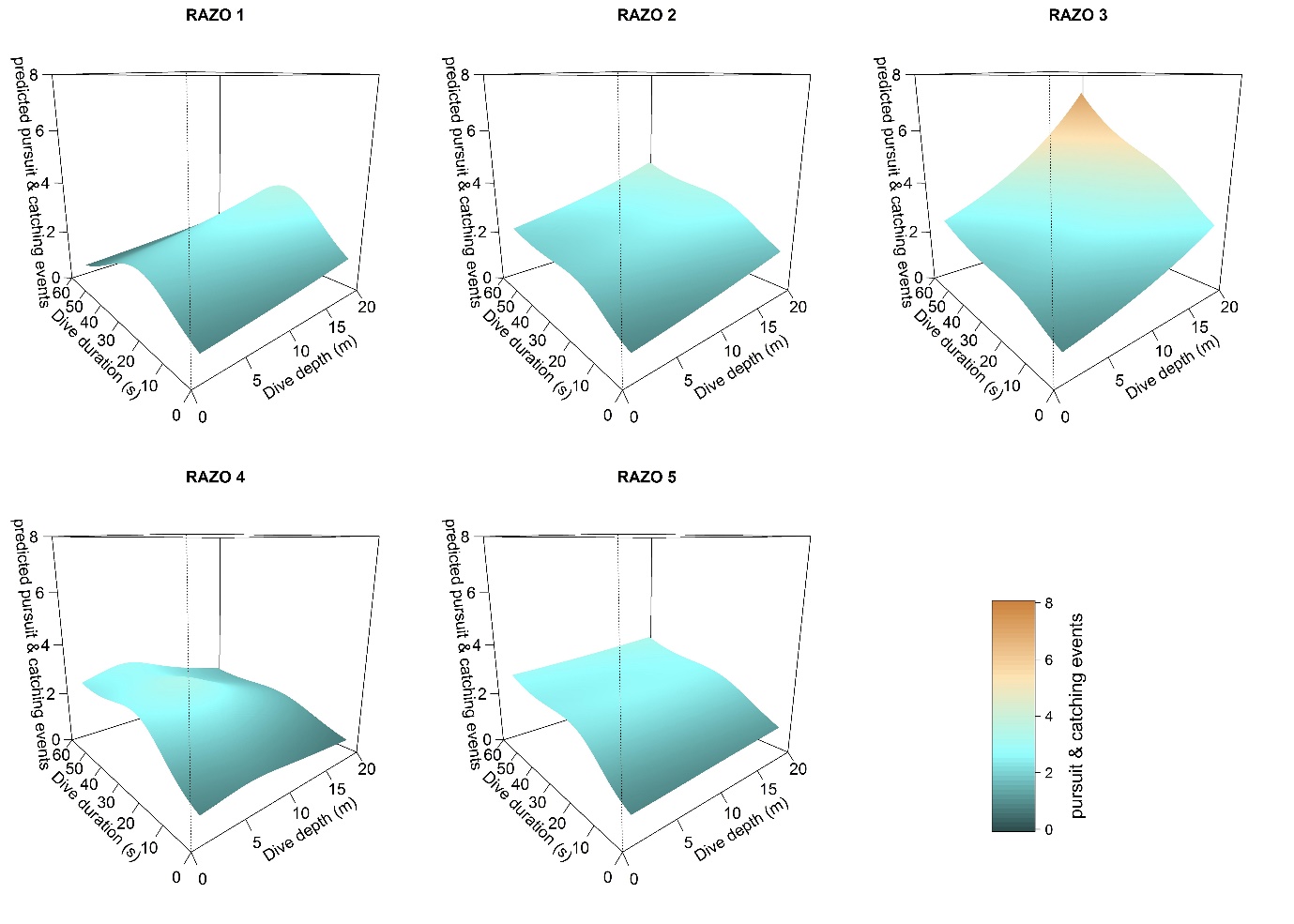


Figure S4: Number of predicted pursuit & catching events given dive duration and depth in razorbills. The distribution of the data used for the model are in figure S2 and S3.
