# Supplementary material for: Taking movement data to new depths: Inferring prey availability and patch profitability from seabird foraging behavior
Source: Ecol Evol. 2017 Oct 25;7(23):10252–65. doi: 10.1002/ece3.3551 (PMC5723613; doi:10.1002/ece3.3551)
Supplement: Supplementary file 2 [file ECE3-7-10252-s002.doc]

##############################################################################

######################### model on common guillemots: catching events

catchingEventsTotCOGU<-read.table("catchingEventsTotCOGU.txt",header=T)

catchingEventsTotCOGU$Animal_ID<-as.factor(catchingEventsTotCOGU$Animal_ID)

catchingEventsTotCOGU$year<-as.factor(catchingEventsTotCOGU$year)

catchingEventsTotCOGU$Class_dive<-as.factor(catchingEventsTotCOGU$Class_dive)

library(mgcv)

model0<-gam(n_catchAttemp~s(time_dive,by=Class_dive,k=3)+s(Depth_dive,by=Class_dive,k=3)+s(Bout_Ntot,bs="re")+Bathymetry:Class_dive+Animal_ID,family=poisson(link = "log"),method="ML", data=catchingEventsTotCOGU)

model2<-gam(n_catchAttemp~te(time_dive,Depth_dive,by=Class_dive,bs="tp",d=2,k=5)+s(Bout_Ntot,bs="re")+Bathymetry:Class_dive+Animal_ID,family=poisson(link = "log"),method="ML", data=catchingEventsTotCOGU)

model3<-gam(n_catchAttemp~s(time_dive,by=Class_dive,k=3)+s(Depth_dive,by=Class_dive,k=3)+ti(time_dive,Depth_dive,by=Class_dive,bs="tp",d=2,k=5)+s(Bout_Ntot,bs="re")+Bathymetry:Class_dive+Animal_ID,family=poisson(link = "log"),method="ML", data=catchingEventsTotCOGU)

anova(model0,model2,test="Chi")

anova(model0,model3,test="Chi")

anova(model2,model3,test="Chi")

AIC(model0,model2,model3)

######################################################################################

################## model on time spent catching during a dive bout - common guillemots

dfCOGU<-read.table("dfCOGU.txt", header=T)

dfCOGU$Animal_ID<-as.factor(dfCOGU$Animal_ID)

dfCOGU$year<-as.factor(dfCOGU$year)

library(mgcv)

preyCOGU <- gam(logtime_catching ~ s(logtime_UW, by=Animal_ID) + Animal_ID, data= dfCOGU)

#######################################################################################

############### model on number of catching events through the water column - razorbills

catchingEventsTotRAZO<-read.table("catchingEventsTotRAZO.txt",header=T)

catchingEventsTotRAZO$Animal_ID<-as.factor(catchingEventsTotRAZO$Animal_ID)

library(mgcv)

model0<-gam(n_catchAttemp~s(time_dive,by=Animal_ID,k=5)+s(Depth_dive,by=Animal_ID,k=5)+s(Bout_Ntot,bs="re")+Animal_ID,family=poisson(link = "log"), data=catchingEventsTotRAZO, method="ML")

model2<-gam(n_catchAttemp~te(time_dive,Depth_dive,by=Animal_ID,bs="tp",d=2,k=5)+s(Bout_Ntot,bs="re")+Animal_ID,family=poisson(link = "log"), data=catchingEventsTotRAZO, method="ML")

model3<-gam(n_catchAttemp~s(time_dive,by=Animal_ID,k=5)+s(Depth_dive,by=Animal_ID,k=5)+ti(time_dive,Depth_dive,by=Animal_ID,bs="tp",d=2,k=5)+s(Bout_Ntot,bs="re")+Animal_ID,family=poisson(link = "log"), data=catchingEventsTotRAZO, method="ML")

anova(model0,model2,model3,test="Chi")

anova(model0,model2,test="Chi")

anova(model0,model3,test="Chi")

anova(model2,model3,test="Chi")

AIC(model0,model2,model3)

modelPop0<-gam(n_catchAttemp~s(time_dive,k=5)+s(Depth_dive,k=5)+s(Bout_Ntot,bs="re")+Animal_ID,family=poisson(link = "log"), data=catchingEventsTotRAZO, method="ML")

modelPop2<-gam(n_catchAttemp~te(time_dive,Depth_dive,bs="tp",d=2,k=5)+s(Bout_Ntot,bs="re")+Animal_ID,family=poisson(link = "log"), data=catchingEventsTotRAZO, method="ML")

modelPop3<-gam(n_catchAttemp~s(time_dive,k=5)+s(Depth_dive,k=5)+ti(time_dive,Depth_dive,bs="tp",d=2,k=5)+s(Bout_Ntot,bs="re")+Animal_ID,family=poisson(link = "log"), data=catchingEventsTotRAZO, method="ML")

anova(modelPop0,modelPop2,modelPop3,test="Chi")

anova(modelPop0,modelPop2,test="Chi")

anova(modelPop0,modelPop3,test="Chi")

anova(modelPop2,modelPop3,test="Chi")

AIC(modelPop0,modelPop2,modelPop3)

######################################################################################

#################### model on time spent catching during a dive bout - razorbills

dfRAZO<-read.table("dfRAZO.txt", header=T)

dfRAZO$Animal_ID<-as.factor(dfRAZO$Animal_ID)

preyRAZO<-gam(time_catching ~ s(time_UW,by=Animal_ID,k=3)+Animal_ID,family=Tweedie(p=1.05, link = "identity"), data=dfRAZO)

summary(preyRAZO)

#######################################################################################

#################### LINEAR MODEL - SPECIES COMPARISON

GeneralModelCOGU<-lm(logtime_catching ~ logtime_UW + Species,data = BothRazo_Cogu)
